# Subclinical Atherosclerosis in Patients with Rheumatoid Arthritis and Low Cardiovascular Risk: The Role of von Willebrand Factor Activity

**DOI:** 10.1371/journal.pone.0130462

**Published:** 2015-08-06

**Authors:** Gorica G. Ristić, Vesna Subota, Toplica Lepić, Dejana Stanisavljević, Branislava Glišić, Arsen D. Ristić, Milan Petronijević, Dušan Z. Stefanović

**Affiliations:** 1 Department of Rheumatology and Clinical Immunology, Military Medical Academy and Medical Faculty of the Belgrade Defence University, Belgrade, Serbia; 2 Institute of Medical Biochemistry, Military Medical Academy, Belgrade, Serbia; 3 Department of Neurology, Military Medical Academy and Medical Faculty of the Belgrade Defence University, Belgrade, Serbia; 4 Institute of Medical Statistics, Belgrade University School of Medicine, Belgrade, Serbia; 5 Department of Cardiology, Clinical Centre of Serbia and Belgrade University School of Medicine, Belgrade, Serbia; VU University Medical Center, NETHERLANDS

## Abstract

**Background:**

To evaluate association between von Willebrand factor (vWF) activity, inflammation markers, disease activity, and subclinical atherosclerosis in patients with rheumatoid arthritis (RA) and low cardiovascular risk.

**Methods:**

Above mentioned parameters were determined in blood samples of 74 non-diabetic, normotensive, female subjects, with no dyslipidemia(42 patients, 32 matched healthy controls, age 45.3±10.0 vs. 45.2±9.8 years). Intima-media thickness (IMT) was measured bilaterally, at common carotid, bifurcation, and internal carotid arteries. Subclinical atherosclerosis was defined as IMT>IMT_mean_+2SD in controlsat each carotid level and atherosclerotic plaque as IMT>1.5 mm. Majority of RA patients were on methotrexate (83.3%), none on steroids >10 mg/day or biologic drugs. All findings were analysed in the entire study population and in RA group separately.

**Results:**

RA patients with subclinical atherosclerosis had higher vWF activity than those without (133.5±69.3% vs. 95.3±36.8%, p<0.05). Predictive value of vWF activity for subclinical atherosclerosis was confirmed by logistic regression. vWF activity correlated significantly with erythrocyte sedimentation rate, fibrinogen, modified disease activity scores (mDAS28–ESR, mDAS28–CRP), modified Health Assessment Questionnaire (p<0.01 for all), duration of smoking, number of cigarettes/day, rheumatoid factor concentration (p<0.05 for all), and anti-CCP antibodies (p<0.01). In the entire study population, vWF activity was higher in participants with subclinical atherosclerosis (130±68% vs. 97±38%, p<0.05) or atherosclerotic plaques (123±57% vs. 99±45%, p<0.05) than in those without. Duration of smoking was significantly associated with vWF activity (β 0.026, p = 0.039).

**Conclusions:**

We demonstrated association of vWF activity and subclinical atherosclerosis in low-risk RA patients as well as its correlation with inflammation markers, all parameters of disease activity, and seropositivity. Therefore, vWF might be a valuable marker of early atherosclerosis in RA patients.

## Introduction

The incidence of cardiovascular diseases (CVD) is higher in patients with rheumatoid arthritis (RA) than in general population [1] and increased carotid intima-media thickness (IMT) has been recommended for the cardiovascular risk stratification in these patients [2, 3]. Chronic inflammation, the basic feature of rheumatoid arthritis (RA), plays a major role in accelerated atherosclerosis in patients with RA through its influence on insulin resistance, lipid status, and atherothrombogenic factors, such as fibrinogen, D-dimer, von Willebrand factor (vWF), and plasminogen activator inhibitor (PAI) [4]. The vWF is considered a reliable marker of endothelial dysfunction/damage, which is an initial step in atherosclerosis [5, 6].

Independent association of vWF with the increased carotid intima-media thickness (IMT) was shown in asymptomatic subjects [7], but limited data are available regarding its relation with subclinical atherosclerosis in RA [8–10], and only two investigations analysed patients without atherosclerotic risk factors [11, 12]. Therefore, the aim of our study was to evaluate association between vWF activity, inflammation markers, disease activity, and carotid IMT in young, non-diabetic, normotensive, female RA patients, with no dyslipidemia.

## Methods

The investigation was designed as a cross-sectional, single-centre study. All participants have signed two copies of a written informed consent to participate in this study (one given to the participant, one kept in the study files). The study protocol and the consent procedure were approved by the Ethics Committee of the Military Medical Academy, Belgrade, Serbia.

### Patients and Controls

The study population included 74 female subjects: 42 RA consecutive patients and 32 healthy controls. Patients fulfilled the American College of Rheumatology revised criteria for RA. Mean disease duration was 7.1±5.4 years. Extra-articular manifestations were present in 11.9%, rheumatoid factor (RF) in 69%, anti-cyclic citrullinated peptide (anti CCP) antibodies in 59.5% of patients. Mean modified disease activity score 28 (mDAS28-ESR) was 3.55±1.36, mDAS28-CRP 3.1±1.27, while mean modified Health Assessment Questionnaire (mHAQ) was 0.45±0.49. Treatment included: low-dose prednisolone in 73.8% (mean 4.0±4.9 years), methotrexate dose of 10.4±2.0 mg/week in 83.3% (mean 3.3±2.8 years), chloroquine in 76.2% (mean 3.7±2.7 years), combined methotrexate/chloroquine therapy in 62% (mean 2.6±1.8 years), and sulfasalazine in 23.8% (mean 2.5±1.8 years) of patients.

Control subjects were matched with RA group regarding age (45.2±9.8 years, range 27–57 vs. 45.3±10.0 years, range 29–58 in RA), menopausal status (31.3% vs. 35.7%), body mass index (25.1±4.1% vs. 24.2±4.5 kg/m^2^), smoking habits, and serum lipid levels.

Subjects with the following conditions were excluded: history of CVD, hypertension, diabetes mellitus, hyperlipidemia, premature menopause, and treatment with biologic drugs and/or steroids >10 mg/day as defined in our previous study [13].

### Laboratory Analyses

The erythrocyte sedimentation rate (ESR) was determined using the modified Westergren method, fibrinogen, glycaemia, total-, high-density, low-density cholesterol, and triglycerides were measured according to the established methods. C-reactive protein (CRP) and rheumatoid factor (RF) were determined by nephelometry, anti-CCP antibodies using ELISA. The vWF activity and PAI-1 were determined by a BC von Willebrand Reagent and Berichrom PAI on coagulation analyzer BCS-XP (Dade Behring/Siemens, Germany) [14], and D-dimer by immunochemistry (D-dimer PLUS reagent, BCS-XP analyzer).

### Carotid Ultrasound

Carotid IMT was measured using a high resolution B-mode (9 MHz) ultrasound (Toshiba SSA370A, Japan). The IMT was defined as the distance between edges of the lumen-intima and the media-adventitia echos, in a plaque-free section. We measured IMT bilaterally, at the levels of common carotid (CCA), carotid bifurcation (BF) and internal carotid artery (ICA), as in the ARIC study [15]. Total of 18 measurements were performed in each subjectand mean values were calculated for all segments (CCA, BF, ICA). Values at any point above mean IMT+2SD of the controls were defined as subclinical atherosclerosis, while atherosclerotic plaque as IMT>1.5 mm [16]. To avoid interobserver variability, all measurements were performed by the same experienced sonographer (TL), blinded for the clinical characteristics of the subjects.

### Statistical Analysis

All findings were analysed in the entire study population and RA group separately. Values were expressed as means±SD or percentages as appropriate. Student’s *t*-test or Mann-Whitney U tests were applied for continuous variables, Chi-square or Fisher’s exact test for categorical variables. Simple and multiple logistic regression analysis were performed to identify predictors of subclinical atherosclerosis. Spearman’s correlation coefficient was used to determine the association between vWF and traditional-, as well as RA-related risk factors for atherosclerosis. All data were analysed using SPSS 15.0, considering a 2-tailed level of p<0.05 as significant.

## Results

### Association of Subclinical Atherosclerosis or Plaques with (Non)Traditional Risk Factors and von Willebrand Factor Activity

According to IMT values in control subjects (CCA_mean_ 0.62±0.09 mm; BF_mean_ 0.80±0.12 mm; ICA_mean_ 0.54±0.08 mm), subclinical atherosclerosis (IMT_mean_+2SD of controls) was determined if IMT was higher than: 0.79 mm at CCA, 1.05 mm at BF, and 0.69 mm at ICA. **Subclinical atherosclerosis** on at least one level was demonstrated in 21.6% of all participants (16/74), in 35.7% of RA patients (15/42), and in 3.1% of controls (1/32) (p<0.01). **Atherosclerotic plaques** (IMT>1.5 mm) were revealed only on carotid BF in 20.3% of all participants (15/74), in 28.6% of RA patients (12/42) and 9.4% in controls 3/32 (p<0.05).

In **the entire study** population, participants with subclinical atherosclerosis, were older than those without (52.7±5.5 vs. 43.2±9.8), had a higher fibrinogen (3.7±0.9 vs. 2.9±0.8), ESR (35.0±24.5 vs. 16.9±15.3) and were more frequently RF positive (81.3% vs. 32.8%) (p<0.01 for all). The univariate regression analysis confirmed association between abovementioned risk factors and presence of subclinical atherosclerosis (Table 1). The same was true for participants with atherosclerotic plaques who were older (53.0±5.7 vs. 43.3±9.7), had a higher fibrinogen (3.8±0.9 vs. 3.0±0.8), ESR (34.4±25.1 vs. 17.6±15.9) and were more frequently RF positive (66.7% vs. 37.3%) than those without plaques (p<0.01 for all) and univariate regression verified this association.

**Table 1:** Univariate and multivariate logistic regression analysis of association between traditional or RA related cardiovascular risk factors with the presence of subclinical atherosclerosis (mean IMT+2SD of the controls) or atherosclerotic plaque (IMT>1.5 mm) in the entire study group and patients with rheumatoid arthritis.

	**All participants**				**Patients with RA**			
	With subclinical atherosclerosis N = 16/74		With plaque N = 15/74		With subclinical atherosclerosis N = 15/42		With plaque N = 12/42	
	β	P	β	P	β	P	β	P
Age (years)	0.145	0.003^a^	0.152	0.003^a^	0.202	0.002	0.154	0.008^a^
ESR (mm/h)	0.045	0.005	0.038	0.010	0.027	ns	0.030	ns
Fibrinogen (g/l)	1.006	0.005	1.061	0.004	0.666	ns	0.852	0.041
RF positive (%)	2.185	0.002	1.213	0.047	1.497	ns	1.063	ns
**vWF (% activity)**	1.290	**0.026**	0.897	ns	1.535	**0.045**	0.263	ns
Smoking habits (cigarettes/day)	0.065	ns	0.062	ns	0.181	0.037^a^	0.072	ns
Rheumatoid arthritis presence	2.846	0.008^a^	1.352	0.049^a^	NA		NA	

β—regression coefficient in the univariate analysis, NA—not applicable, vWF—Von Willebrand factor, RF—rheumatoid factor.

^a^ Also significant in multiple regression analysis, which included parameters showing significant difference in the univariate analysis (p<0.01 for all, except for smoking habits and subclinical atherosclerosis, where p<0.05)

There was no significant difference between the groups regarding all lipid parameters and blood glucose levels. In the multiple regression analysis only age and RA itself were independent risk factors for subclinical atherosclerosis and plaque (p<0.01).

Subjects with subclinical atherosclerosis or plaque had significantly higher vWF activity than those without (130±68 vs. 97±38, p = 0.026; for plaque 123±57 vs. 99±45, p = 0.028) and this association was verified in the logistic regression analysis (Table 1). Among other haemostatic factors, D-dimer was higher in subjects with subclinical atherosclerosis or plaques than those without, but the difference was not significant. No difference was present regarding PAI-1 activity.

In participants with subclinical atherosclerosis or plaque, all smoking parameters were higher, but statistical significance was not reached. In subjects with and without subclinical atherosclerosis the following smoking habits were noted: smokers 68.8% vs. 55.2%, duration of smoking 22±8.8 vs. 19.4±8.2 years, cigarettes per day 20±7.7 vs. 15.4±8.4. In subjects with and without atherosclerotic plaques parameters regarding smoking were as follows: smokers 66.7% vs. 55.9%, duration of smoking 22±9.1 vs. 19.6±8.2 years, cigarettes per day 20±9.4 vs. 15.6±7.9. Univariate regression analysis did not reveal significant association of subclinical atherosclerosis/plaques and smoking habits. Only duration of smoking was close to significant association with subclinical atherosclerosis (β 0.182, p = 0.060).

In the univariate analysis of the impact of smoking habits on vWF activity, statistical significance was obtained only for the duration of smoking (β 0.026, p = 0.039). There was no significant difference in vWF activity between smokers (30/74) and non-smokers (44/74): 106±53 vs. 102±45, p = 0.895. Importantly, vWF activity was significantly higher in smokers with subclinical atherosclerosis (6/30) than in those without (24/30) (166±82 vs. 91±31, p = 0.006). **RA patients** with subclinical atherosclerosis, compared to those without, were older (53.2±5.4 vs. 40.9±9.3 years) and have smoked more cigarettes/day (20±7.7 vs. 12.6±6.4) (p<0.01 for both), as confirmed by univariate analysis (Table 1). Multivariate analysis revealed that only smoking (p<0.05) was an independent risk factor for subclinical atherosclerosis. RA patients with atherosclerotic plaques, compared to those without, were older (52.7±6.1 vs. 42.4±9.7 years) and had a higher fibrinogen (3.9±0.9 vs. 3.2±0.9) (p<0.05 for both), as verified in the univariate analysis. Multivariate analysis demonstrated that only age had predictive value for plaque (p<0.01).

Patients with subclinical atherosclerosis were more frequently RF positive but this was not statistically significant (86.7% vs. 59.3%, p = 0.065) and there was no predictive value in simple regression model (p = 0.079). Patients without atherosclerosis had longer RA duration than those with atherosclerosis (8.2±5.8 vs. 5.2±4.2 years, p = 0.085), but duration of their combined methotrexate/chloroquine therapy was more than twice longer (3.0±1.9 vs. 1.4±0.5 years, p = 0.065). In regression model a negative association was found between atherosclerosis and duration of this therapy (p = 0.069).

In the **RA group**, patients with subclinical atherosclerosis had significantly higher vWF activity compared to those without (134±69 vs. 95±37, p = 0.024) and this association was validated by the logistic regression. Patients with atherosclerotic plaques had higher vWF activity but the difference was not significant. No significant difference between the groups was present regarding D-dimer and PAI-1 levels.

### Correlation of von Willebrand Factor Activity and Other Haemostatic Factors with Clinical, Laboratory Features, and Anti-Rheumatic Treatment in RA Patients

There was a significant correlation of vWF activity with age, duration of smoking, number of cigarettes/day, markers of inflammation, RF concentration and anti-CCP antibodies, and all parameters of RA activity (Table 2). However, a negative correlation of vWF activity with anti-inflammatory treatment was non-significant. Fibrinogen correlated with disease activity, other markers of inflammation, and RF concentration. D-dimer correlated well with markers of inflammation and disease activity.

**Table 2:** Correlation of haemostatic factors with clinical, laboratory features, and treatment in patients with rheumatoid arthritis.

Clinical and laboratory features of patients with rheumatoid arthritis	vWF	Fibrinogen	D-dimer	PAI-1
Age (years)	0.490***	0.272	0.023	0.056
Smoking (years)	0.581*	0.280	-0.009	-0.010
Smoking (cigarettes/day)	0.536*	0.123	-0.545*	0.407
Erythrocyte sedimentation rate(mm/h)	0.498***	0.751***	0.376*	0.109
Fibrinogen (g/l)	0.466**	/	0.124	0.009
C reactive protein (mg/l)	0.247	0.636***	0.463**	0.193
Rheumatoid factor (IU/ml)	0.421*	0.440*	0.162	0.100
Anti-cyclic citrullinated peptide antibodies (IU/ml)	0.586**	0.146	0.148	0.084
Visual analogue scale general health patient (mm)	0.439**	0.465 **	0.215	0.107
No of swollen joints, 28 assessed	0.365*	0.523 ***	0.245	0.242
No of tender joints, 28 assessed	0.476***	0.527***	0.271	0.137
Modified disease activity score (mDAS28-ESR)	0.552***	0.778***	0.377*	0.007
Modified disease activity score (mDAS28-CRP)	0.446**	0.672***	0.418**	0.009
Modified Health Assessment Questionnaire	0.406**	0.450**	0.300	0.114
Duration of methotrexate/chloroquine combined therapy (years)	-0.18	-0.19	0.01	0.06
Methotrexate therapy—average weekly dose (mg/week)	-0.32	0.06	0.14	-0.11

All values are Spearman's correlation coefficients.

*p<0.05;

** p<0.01;

*** p<0.001

vWF—von Willebrand factor, PAI—plasminogen activator inhibitor.

## Discussion

Our study demonstrated **significant association of vWF activity and the presence of subclinical atherosclerosis** inyoung, non-diabetic, normotensive, female RA patients, with no dyslipidemia. We have also shown its significant **correlation with inflammation markers, all parameters of disease activity, RF concentration, and anti-CCP antibodies**.

Only few studies assessed relationship between **haemostatic factors and subclinical atherosclerosis in RA** [8–12] and two of them analysed RA patients without atherosclerotic risk factors [11, 12]. Our results are consistent with the report by Daza et al. [11] who evaluated very similar RA group. Södergren et al. [10] revealed significant correlation between CCA-IMT and vWF activity in RA patients and in multiple regression analysis vWF activity was the best predictor for increased IMT. However, their study group was not free from confounding atherosclerotic risk factors.

The major pitfalls in interpreting vWF activity impact on cardiovascular risk arise from the fact that its levels are strongly influenced by age. Poor arterial compliance associated with ageing causes increased endothelial vWF secretion which than contributes to the advanced cardiovascular risk [17]. We also found highly significant correlation of vWF activity with age in RA patients, which was probably the reason for the loss of independent predictive value of vWF activity for subclinical atherosclerosis in multiple regression models. Recently, Fan et al. [18] reported significant correlation of vWF with arterial stiffness (assessed by pulse wave velocity) that also disappeared after adjustment for age. Although arterial stiffness represents potentially reversible atherosclerotic changes, while increased carotid IMT and plaque signify irreversible lesions, both are considered surrogate markers of atherosclerosis [16, 19, 20]. According to these results, vWF might be important in very early stages of atherosclerosis, as well as in the later subclinical stage with increased IMT and formation of plaques. Both stages are facilitated by chronic inflammation in RA.

We obtained significant positive correlation of vWF activity with ESR, fibrinogen, and all parameters of disease activity. It was previously demonstrated that several inflammatory cytokines, including tumour necrosis factor (TNF)-α, interleukin (IL)-8, and IL-6 stimulate vWF release from endothelial cells in a dose-dependent manner [21]. Moreover, TNF-α and IL-6 are the key inflammatory cytokines in RA, which could explain correlation between vWF and RA activity. Due to its association with inflammation vWF may be considered as acute phase protein and pathophysiological link between RA activity and endothelial damage.

The association of vWF with inflammation was also reflected by the negative correlation of vWF activity with duration of anti-inflammatory therapy. However, these correlations were not statistically significant, probably due to the cohort size.

In contrast to our study, Södergren et al. found significant relation between disease activity, tPA, and PAI-1 mass, but not with vWF activity [10]. However, their study group was not free from traditional atherosclerotic risk factors.

Wållberg-Johnson et al. also found correlation of vWF, D-dimer, and PAI-1 with ESR, while PAI-1 and D-dimer correlated with accumulated disease activity [22]. Foster et al. demonstrated significantly higher levels of vWF in RA group, but revealed no correlation between vWF and inflammation or disease activity [23, 24]. Yet, both studies used vWF concentration instead its activity and included elderly patients with cardiovascular risk factors.

However, Veselinovic et al. [12] were not able to confirm predictive value of vWF activity for IMT changes over time, although RA patients had significantly higher vWF activity in comparison with controls at initial and repeated measurements. Paradoxically, predictive value for differences in IMT over time was also not shown for age and lipid parameters. These findings could be explained by a complex interplay between traditional risk factors, inflammation-mediated metabolic, atherothrombogenic processes, and anti-inflammatory therapy, which all have influence on atherosclerotic process in RA patients.

Unexpectedly, we found significant correlation of vWF activity with RF concentration as well as with anti-CCP antibodies. Tomasson et al. [25] have revealed that seropositivity was associated with increased cardiovascular mortality even in general population without joint symptoms. In RA patients, seropositivity is frequently associated with extra-articular manifestations including rheumatoid vasculitis. Importantly, these patients have significantly higher plasma levels of vWF than patients without vasculitis and the normal subjects.

Among **traditional atherosclerotic risk factors** in the entire study population there was no significant difference between the groups with and without subclinical atherosclerosis/plaque, regarding lipids, blood glucose levels, and smoking habits, although all smoking parameters were higher in participants with atherosclerosis/plaque. In the univarate analysis smoking duration was the most relevant factor significantly associated with vWF activity. This result is in concordance with the previous reports that smoking increased levels of vWF [26, 27] while smoking cessation has an opposite effect [28]. There was no significant difference in vWF activity between smokers and non-smokers. However, vWF activity was significantly higher in smokers with subclinical atherosclerosis, than in those without. This finding could be a consequence of a cumulative effect of chronic inflammation and smoking that potentiate each other in advancing atherosclerosis. Importantly, in RA patients we found that smoking duration and number of cigarettes/day significantly correlate with vWF activity. On the other hand, for subclinical atherosclerosis smoking even outweighs the importance of age in multivariate analyses. Among studies that included smokers [9, 22, 29, 30] only two reported significant impact of smoking [10, 31].

Among **RA-related atherosclerotic risk factors**, seropositivity and disease duration of more than 10 years are considered the most important [32]. In our study, patients with subclinical atherosclerosis were more often RF positive than those without, but its predictive value was not significant. Association with disease duration was also not confirmed. Patients without subclinical atherosclerosis even had RA longer than those with atherosclerosis, but this group had more than twice longer duration of combined methotrexate/chloroquine therapy. Importantly, the number of patients on combined therapy was higher in group with normal IMT values. Accordingly, we found negative association between the presence of subclinical atherosclerosis and duration of this therapy, but statistical significance was not reached. Our results are consistent with previous studies in which methotrexate treatment decreased IMT [9, 13, 33], confirming the importance of inflammatory burden for accelerated atherosclerosis in RA [34]. Sustained control of inflammation and reduced disease activity was demonstrated to also reduce the risk for cardiovascular events in a recentlarge-scale prospective study [35, 36].

## Conclusions

**Significant association of vWF activity and subclinical atherosclerosis** in RA patients with low cardiovascular risk as well as its **correlation with inflammation markers and disease activity** implicates a pathophysiological link between RA and endothelial damage. Correlation of vWF and **RF** may be a manifestation of a process contributing to the accelerated atherosclerosis in RA. Therefore, **vWF activity might be a valuable marker of early atherosclerosis in RA patients** who may benefit from prevention strategies.

## Supporting Information

S1 Table Database underlying the findings in the manuscript. (XLSX)
